# The Disruption of a Nuclear Export Signal in the C-Terminus of the Herpes Simplex Virus 1 Determinant of Pathogenicity UL24 Protein Leads to a Syncytial Plaque Phenotype

**DOI:** 10.3390/v15091971

**Published:** 2023-09-21

**Authors:** Carmen Elena Gonzalez, Nawel Ben Abdeljelil, Angela Pearson

**Affiliations:** Centre Armand-Frappier Santé Biotechnologie, Institut National de la Recherche Scientifique, Laval, QC H7V 1B7, Canada

**Keywords:** herpes simplex virus, UL24, nuclear export signal, syncytia

## Abstract

*UL24* of herpes simplex virus 1 (HSV-1) has been shown to be a determinant of pathogenesis in mouse models of infection. The N-terminus of UL24 localizes to the nucleus and drives the redistribution of nucleolin and B23. In contrast, when expressed alone, the C-terminal domain of UL24 accumulates in the Golgi apparatus; its importance during infection is unknown. We generated a series of mammalian expression vectors encoding UL24 with nested deletions in the C-terminal domain. Interestingly, enhanced nuclear staining was observed for several UL24-deleted forms in transient transfection assays. The substitution of a threonine phosphorylation site had no effect on UL24 localization or viral titers in cell culture. In contrast, mutations targeting a predicted nuclear export signal (NES) significantly enhanced nuclear localization, indicating that UL24 is able to shuttle between the nucleus and the cytoplasm. Recombinant viruses that encode UL24-harboring substitutions in the NES led to the accumulation of UL24 in the nucleus. Treatment with the CRM-1-specific inhibitor leptomycin B blocked the nuclear export of UL24 in transfected cells but not in the context of infection. Viruses encoding UL24 with NES mutations resulted in a syncytial phenotype, but viral yield was unaffected. These results are consistent with a role for HSV-1 UL24 in late cytoplasmic events in HSV-1 replication.

## 1. Introduction

Herpes simplex virus 1 (HSV-1) is a member of the *Simplexvirus* genus within the *Herpesviridae* Family. HSV-1 is the causative agent of several diseases, including orofacial herpes, herpes keratitis, genital herpes, and herpes encephalitis. There are risks of serious complications in newborns and immunocompromised individuals [1]. *UL24* of HSV-1 is conserved among all *Herpesviridae* members [2]. UL24 is a late viral protein, highly basic, and with a molecular weight of 29.5 KDa [3]. The N-terminal portion is conserved between *Herpesviridae* orthologs [4,5]. Previous studies revealed the importance of several conserved amino acids in the N-terminus of UL24 for nucleolar modifications induced by HSV-1 as well as for pathogenesis in a mouse model of ocular infection [6,7]. In contrast, the C-terminal domain of UL24 shows low sequence conservation. A putative bipartite nuclear localization signal (NLS) has been identified in the C-terminus, although its functionality has yet to be demonstrated [8]. UL24 is important for efficient viral replication in cell culture; UL24-deficient viruses exhibit decreased viral titers and a syncytial plaque phenotype whereby the plasma membranes of the infected cells fuse together [4,9,10]. UL24 is an important determinant of neuropathogenesis. In a mouse model of ocular infection, a UL24-deficient virus exhibits reduced dissemination from the cornea to neurons of the trigeminal ganglia [11]. There is an approximate 4 log_10_ reduction in titers in the trigeminal ganglia and a decrease in efficient viral reactivation from latency in an explant model [12]. UL24 has profound effects on nucleolar architecture during infection. It induces the redistribution of the major nucleolar proteins nucleolin and B23 [13,14], which function in ribosome biogenesis and maturation [15]. UL24 also affects events in the cytoplasm. It inhibits the DNA sensing component of the cellular antiviral response by blocking the nuclear translocation of NFκB [16]. In addition, infection with a virus that does not express the UL24 protein, such as UL24X, results in the mislocalization of at least two of the viral glycoproteins involved in membrane fusion: gB and gD. These viral glycoproteins appear to colocalize with the cytoskeleton, and this association is greatly diminished in the absence of UL24 late in the replication cycle [17]. This effect may contribute to the syncytial plaque phenotype associated with several *UL24* mutations. The combination of nuclear and cytoplasmic/membrane-related activities suggests that UL24 is a multifunctional protein. Studies on the subcellular localization of UL24 are consistent with this model. In both cellular fractionation and confocal microscopy experiments, UL24 is associated with both the nuclear and cytoplasmic compartments of HSV-1-infected cells [3]. Similarly, in transfected cells, UL24 is detected in both compartments. Furthermore, in transient transfection assays, the N-terminus of UL24 accumulates in the nucleus, while the C-terminus of the protein accumulates in the Golgi apparatus [8]. These observations suggest that UL24 contains appropriate targeting signals that regulate its intracellular distribution and trafficking.

The import and export of proteins to and from the nucleus occur through protein transporters, importins, and exportins, which recognize specific NLS and nuclear export signals (NESs) [18,19]. Exportin 1 (CRM-1) is a common nuclear export receptor that plays a role in the export of proteins containing an NES [20]. CRM-1-dependent nuclear export can be specifically inhibited by the drug leptomycin B (LMB), which blocks the NES binding site on CRM-1 [21]. The NES recognized by CRM-1 is a short peptide sequence enriched in hydrophobic amino acids originally defined by the consensus sequence Ø^1^X_2-3_Ø^2^X_2-3_Ø^3^XØ^4^, where Ø represents hydrophobic amino acids, and X is any amino acid [22]; however, the classification of consensus NESs has been updated based on the NES-CRM-1 interaction: the human immunodeficiency virus type 1 (HIV-1) Rev class NES (Ø^0^Ø^1^XØ^2^XXØ^3^XØ^4^) and the PKI class NES (Ø^0^XXØ^1^XXXØ^2^XXØ^3^XØ^4^). These newer consensus NESs have five hydrophobic residues important for the NES/CRM-1 interaction rather than four as is found in the original consensus NES [23]. Several studies have demonstrated the presence of classic CRM-1-dependent NES in viral proteins, such as the HIV-1 Rev protein [24], the bovine immunodeficiency virus (BIV) Rev protein [25], the Kaposi’s sarcoma-associated herpesvirus (KSHV) LANA2 protein [26], the Jembrana disease virus Rev protein [27], and the HSV-1 UL3 and UL47 proteins [28,29]. There are also “non-consensus” NESs, which are important for the localization of proteins in the cytoplasm and have been identified for several viral proteins, such as the feline immunodeficiency virus (FIV) and equine infectious anemia virus (EIAV) Rev proteins [30], the porcine reproductive and respiratory syndrome virus (PPRSV) N-protein [31], the Zika virus NS3 protein [32], and the hepatitis C virus (HCV) core protein [33]. Such NESs differ in the length and sequence of the amino acid stretches between the hydrophobic positions. An updated CRM1-NES database is available [34].

In this study, we performed mutational analyses to test the hypothesis that there are residues in the C-terminal domain of UL24 that are important for its functions in the cytoplasm and for viral replication. As evidenced by our results, we identified a functional NES that influences plaque phenotype in cell culture.

## 2. Materials and Methods

### 2.1. Cell Culture and Viruses

All cell lines were maintained at 37 °C in a humidified incubator with 5% CO_2_. COS-7 (ATCC CRL-1651) (ATCC, Virginia, USA) and Vero cells (ATCC CCL-81) (African green monkey kidney fibroblasts), were cultured in Dulbecco’s modified Eagle’s medium (DMEM) supplemented with 5% newborn calf serum (NCS) (Wisent, St-Bruno, QC, Canada) and antibiotics (50 U penicillin ml^−1^ and 50 μg streptomycin ml^−1^) (Thermo Fisher Scientific—Gibco, Ottawa, ON, Canada). Human epithelial HeLa (ATCC CCL-2) cells were grown in DMEM supplemented with 8% fetal bovine serum (FBS) (Wisent, St-Bruno, QC, Canada) and antibiotics. The virus vHA-UL24 is described elsewhere [14]. All viruses were propagated on Vero cells grown in DMEM supplemented with 5% NCS and antibiotics. For plaque morphology assays, the infected cells were propagated as described previously [6]. The plaque morphology produced by the recombinant viruses was visualized using an inverted phase contrast Nikon Elipse TE 2000-U microscope (10× objective) equipped with a CoolSNAP HQ camera (Nikon Canada, Mississauga, ON, Canada). Figures based on the TIFF files of images were assembled using Adobe Photoshop. This project was covered by the biosafety certificate #2008-19 issued by the Institutional Biosafety Committee of INRS, Centre Armand-Frappier Santé Biosécurité (original approval date 2008, subject to review and renewal annually). 

### 2.2. Plasmid Construction

Vectors for producing truncated forms of UL24 were generated via PCR using sets of primers containing stop codons in all three open reading frames (ORFs) of UL24, and pKOSHA-UL24 [14] as the template. The primer sequences used for cloning are listed in Table 1.

The PCR products were ligated into the plasmid pBluescript SK+ (Stratagene, California, USA) using T4 DNA ligase (New England Biolabs, Massachusetts, USA), and subsequently excised with BglII and SalI (New England Biolabs). The obtained inserts were each ligated to the vector pKOSHA-UL24 that had also been digested with BglII and SalI. The various versions of *HA-UL24* were excised using Pfl23II (Thermo Fisher Scientific—Fermentas, Waltham, MA, USA) and SalI and swapped into the mammalian expression vector pLBPFl-HA-UL24 [8] that had been digested with the same restriction enzymes. Thus, the final expression vectors encode a hemagglutinin (HA) tag in the N-terminal domain of UL24. Point mutations in the *UL24* gene were introduced via PCR-based site-directed mutagenesis using the vector pAG5 [35] as the template. The corresponding primer sequences used to generate the point mutations are listed in Table 2. Mutated sequences were excised from the vector pAG5 and ligated into the mammalian expression vector pLBPFl-HA-UL24 using the enzymes Bst1107I (Thermo Fisher Scientific—Fermentas, Waltham, MA, USA) and SalI. All clones were verified by DNA sequencing, which was carried out by the McGill University and Genome Quebec Innovation Centre.

### 2.3. Generation of BACmids and Recombinant Viruses

Recombinant viruses were generated via a two-step Red recombination using a bacterial artificial chromosome (BAC)-based system as described previously [36,37]. The *Escherichia coli* strain GS1783, carrying a BAC containing the infectious full-length genome of HSV-1 strain KOS was obtained from Donald M. Coen (Harvard Medical School, Boston, MA, USA) and was used as the starting material [38]. Briefly, to generate point mutations or insert small sequences into the *UL24* coding sequence, mutagenic oligonucleotides were used to amplify the kanamycin resistance gene and insert the mutations in the viral genome. The primer sequences used for mutagenesis are listed in Table 3. Two independent isolates named (1) and (2) were obtained for each recombinant BAC. First, BAC_KOS was mutated to encode UL24 with an N-terminal HA tag, resulting in BAC_KOS HA-UL24 (1) and (2). Subsequently, from this BAC, mutated versions of UL24 containing the mutations T195A and L253A/F254A were produced to generate BAC_KOS HA-UL24 T195A (1) and (2) and BAC_KOS HA-UL24 L253A/F254A (1) and (2), respectively. The rescue viruses were generated using BAC_KOS HA-UL24 L253A/F254A (1) and (2) as templates into which the wild-type sequence was reinserted into the genome. The BAC DNAs were extracted using the NucleoBond PC100 kit (Macherey-Nagel, Düren, Germany), following the manufacturer’s instructions. BAC DNAs were analyzed via PCR and sequencing to confirm the removal of the *KAN^R^* gene and the insertion of the desired mutation in *UL24*. Each BAC DNA was subjected to restriction enzyme digestion with *EcoR*V (New England Biolabs, Ipswich, MA, USA) and analyzed via agarose gel electrophoresis to ensure there were no major genomic rearrangements. To produce an infectious virus, BAC DNAs were transfected into Vero cells using Lipofectamine (Thermo Fisher Scientific—Life Technologies, Waltham, MA, USA) according to the manufacturer’s instructions. Recombinant viruses were purified by limiting dilution two times to ensure that the viral stock represented a single clone. The *UL24* gene of each recombinant virus was sequenced to ensure the absence of undesired mutations.

### 2.4. Viral Replication Curves

Viral yield was assessed using one-step replication assays as described previously [6]. 

### 2.5. Transient Transfections, Virus Infection, and Western Blotting

For transient transfections, 3.5 × 10^5^ COS-7 cells were seeded per well in six-well plates. The following day, cells were transfected with 1.5 μg of plasmid DNA using the FuGene 6 transfection reagent (Promega, Madison, WI, USA) according to the manufacturer’s instructions. For virus infection, 1 × 10^6^ Vero cells were seeded per well in six-well plates. The following day, cells were mock-infected or infected with the indicated virus at a multiplicity of infection (MOI) of 10. Then, 48 h post-transfection or 18 h post-infection (hpi), cells were washed with Dulbecco’s phosphate buffered saline 1X (DPBS) and then lysed in the presence of RIPA lysis buffer (50 mM Tris, 1% Triton X-100, 0.5% deoxycholic acid, 0.1% sodium dodecyl sulfate (SDS), and 500 mM NaCl) in which a complete protease inhibitor cocktail tablet was dissolved (Millipore Sigma—Roche, Darmstadt, Germany). Cell lysates were fractionated on a 12.5% SDS–polyacrylamide gel, and proteins were transferred to a polyvinylidene fluoride membrane (Millipore Sigma—EMD Millipore, Darstadt, Germany). The primary antibodies used for immunoblotting were as follows: mouse monoclonal anti-HA (Covance, Burlington, NC, USA), mouse monoclonal anti-TK (W. Summers, Yale University, New Haven, CT, USA), mouse monoclonal anti-gD (Abcam, Waltham, MA, USA), and rabbit polyclonal anti-α Tubulin (Abcam). Secondary antibodies were as follows: goat anti-mouse peroxidase-conjugated IgG (Jackson ImmunoResearch, West Grove, PA, USA) and goat anti-rabbit peroxidase-conjugated IgG (Bethyl Laboratories, Montgomery, TX, USA). Detection was carried out via enhanced chemiluminescence using the Immun-Star HRP Substrate Kit (Bio-Rad, St-Laurent, Canada) and Hyperfilm ECL (GE Healthcare, Mississauga, ON, Canada).

### 2.6. Confocal Microscopy

COS-7, Vero, or HeLa cells were seeded on coverslips in 24-well plates and transfected or infected as indicated in the text. Cells were fixed 48 h post-transfection, or at 9 and 18 hpi, in 2% (*v*/*v*) paraformaldehyde for 10 min. For leptomycin B (LMB) treatment, 22 h post-transfection or 8 hpi, media were replaced with media containing LMB (Millipore Sigma, Darmstadt, Germany) at a final concentration of 10 ng/mL, 20 ng/mL, or 25 ng/mL for 5 h. Cells were then fixed as described above. Immunofluorescence staining was performed as described previously [17]. The following primary antibodies were used: rat monoclonal anti-HA high affinity (Millipore Sigma—Roche, Darmstadt, Germany), mouse monoclonal anti-nucleolin (Santa Cruz Biotechnology, Dallas, TX, USA), and mouse monoclonal anti-cyclin β1 (Abcam, Waltham, MA, USA). Secondary antibodies were as follows: goat anti-rat IgG Alexa Fluor 488, goat anti-mouse IgG Alexa Fluor 568, and goat anti-mouse IgG Alexa Fluor 488 (Thermo Fisher Scientific—Life Technologies, Waltham, MA, USA). Cells were visualized using a Zeiss LSM780 confocal system equipped with a 30 mW 405 nm diode laser, 25 mW 458/488/514 argon multiline laser, 20 mW DPSS 561 nm laser, and 5 mW HeNe 633 nm laser mounted on Zeiss Axio Observer Z1 (63X objective, N.A. 1.4) and operated with ZEN 2011 software (Zeiss Canada, Toronto, ON, Canada). Images were processed using Adobe Photoshop. Immunofluorescence imaging was carried out at the INRS Centre Armand-Frappier Santé Biotechnologie confocal microscopy facility. The quantification of nuclear versus cytoplasm staining in COS-7 cells was carried out using the ZEN 2011 software (Zeiss). For each condition, the nuclear–cytoplasmic (N/C) ratio of the mean intensity of fluorescence was determined from 30 cells over 3 independent experiments. Statistical significance was evaluated using the one-way ANOVA test (** *p* < 0.0003); the alpha value was subjected to the multiple-comparison Bonferroni correction.

## 3. Results

### 3.1. Residues in the C-Terminal Domain of UL24 Are Important for the Cytoplasmic Accumulation of UL24

**Figure 1:**
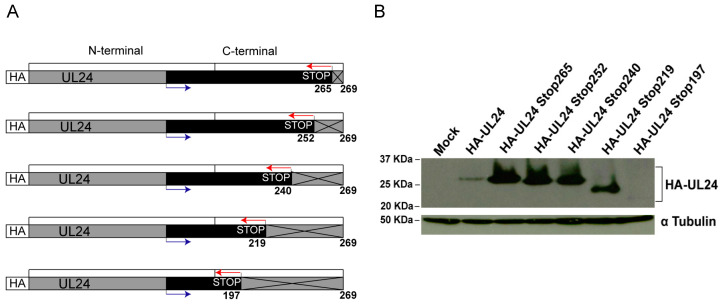
Deletion mutations targeting the C-terminal domain of HSV-1 UL24: (**A**) The schematic representation of the deletion series created for the C-terminal domain of UL24. The HA tag is represented with a white box, and the UL24 amino acid sequence is shown with the gray and black boxes to the right. The numbers below the boxes represent UL24 amino acids. The black box represents the amino acids corresponding to the fragment of *UL24* that was amplified via PCR to construct the respective plasmid, with the position of the primers used for amplification shown in blue and red. Stop codons were introduced into the three ORFs to block translation after aa 265, 252, 240, 219or 197. The crossed lines in the gray box represent the deleted regions of UL24. (**B**) Western blot analysis showing the expression of the full-length and truncated HA-UL24 proteins 48 h post-transfection. Cell lysates of COS-7 cells transfected with a plasmid encoding wild-type HA-UL24 or encoding HA-UL24 with the indicated deletion were analyzed using Western blotting with an anti-HA antibody (**top panel**). The blot was subsequently stripped and incubated with an antibody against α Tubulin, which served as a loading control (**bottom panel**). The sizes of molecular weight markers are indicated to the left of the panels.

To determine the importance of C-terminal residues of UL24 in the subcellular localization and function of the protein, a panel of UL24 truncation mutations was constructed. A diagram showing the deletion strategy is presented in Figure 1A. Transient expression vectors were constructed for UL24 forms in which the translation of the protein is stopped prematurely at amino acids (aa) 265, 252, 240, 219, or 197 through the insertion of stop codons in all three reading frames. The expression of the different versions of UL24 was confirmed with Western blot analysis (Figure 1B). Lower levels of expression were observed for wild-type HA-UL24 and mutant HA-UL24Stop197; however, all constructs expressed proteins of the expected size.

**Figure 2:**
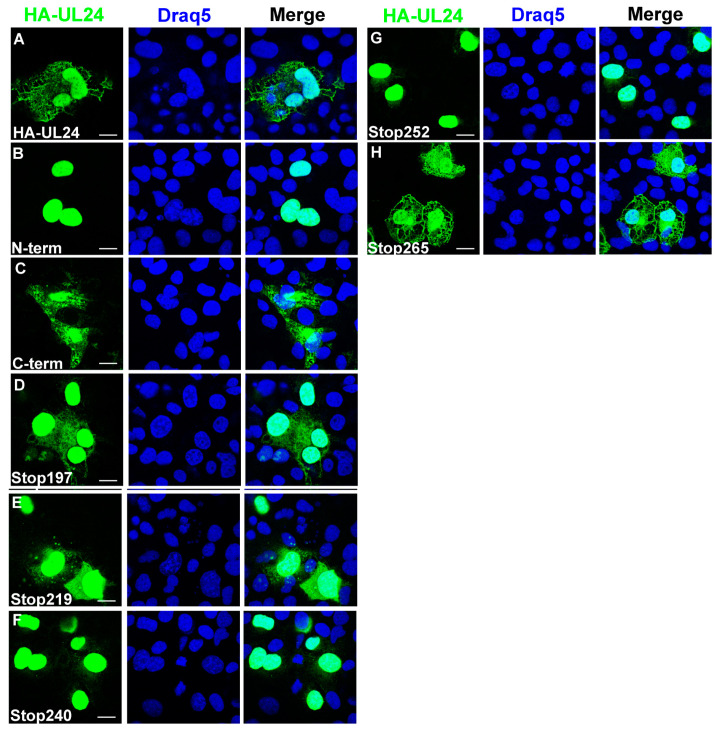
Deletions in the C-terminus of UL24 lead to nuclear sequestration of the protein. COS-7 cells were transiently transfected with plasmids encoding the indicated forms of UL24 protein: (**A**–**C**) Confocal images showing the localization of wild-type HA-UL24 and the N-terminal and C-terminal domains. (**D**–**H**) The localization of HA-UL24 variants with C-terminal truncations. The amino acid indicated represents the last one before the inserted stop codon. HA-UL24 was detected via indirect immunofluorescence using a monoclonal antibody directed against HA (green). Nuclei were stained with Draq5 (blue). Merged images are shown in the right-hand panels. Scale bars represent 10 μm.

Microscopy studies have shown that wild-type HA-UL24 (aa 1-269) is present in both the nuclear and cytoplasmic compartments [14]. Moreover, the N-terminal domain of HA-UL24 (aa 1-192) localizes to the nucleus, while the C-terminal domain of HA-UL24 (aa 190-269) is located in the cytoplasm [8]. The nuclear localization of the N-terminus of UL24 may be related to a nucleolar localization signal within the first 60 amino acids of the protein that we previously identified [8]. We investigated the subcellular localization of the different C-terminally truncated versions of UL24 via confocal microscopy. COS-7 cells were transiently transfected with the plasmids encoding the truncated forms of UL24. We observed that HA-UL24Stop197, HA-UL24Stop219, and HA-UL24Stop265 all localized to the nucleus and the cytoplasm similar to the staining observed for HA-UL24. In contrast, HA-UL24Stop240 and HA-UL24Stop252 were predominantly nuclear (Figure 2B–F). The results for HA-UL24Stop197 and HA-UL24Stop219 were surprising since cytoplasmic accumulation for longer forms was lost. This may reflect the impact of these smallest deleted forms being below the exclusion limit for passive diffusion across the nuclear pore. As well, for HA-UL24Stop197, the disruption of a known phosphorylation site at T195 might be involved. Our results show that the C-terminal domain of UL24 contains residues in the region between aa 241 and 265 that are important for the accumulation of the protein in the cytoplasm.

### 3.2. Identification of an NES in the C-Terminus of HSV-1 UL24

**Figure 3:**
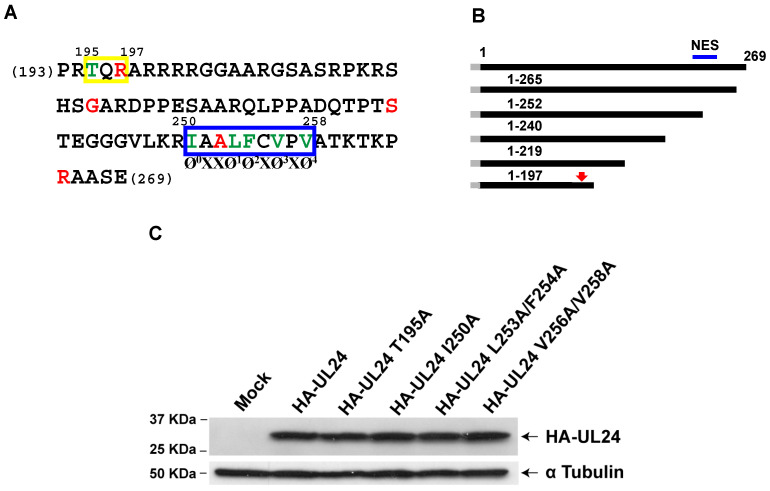
Site-directed mutagenesis targeting the C-terminal domain of HSV-1 UL24: (**A**) The primary sequence of the C-terminal portion of UL24 (aa 193–269). Amino acids in red represent the last amino acid expressed for each of the various truncated forms of UL24. The yellow rectangle delineates a putative phosphorylation site (aa 195–197) identified using the MotifScan software. The blue rectangle delineates a possible NES sequence (aa 250–258) identified through comparison with known viral NESs; the positions of the hydrophobic amino acids thought to form the non-classical NES in UL24 are shown below the blue rectangle: Ø represents a hydrophobic residue, and X indicates any residue. Amino acids in green were replaced with alanines to test their importance in the subcellular localization of the protein. (**B**) Graphic representation showing the position of the phosphorylation site (red arrow) and predicted NES (blue line) in the HSV-1 UL24 full-length protein and truncated versions. The HA tag is represented with a gray box. (**C**) Western blot analysis showing the expression of the wild-type and substituted forms of HA-UL24 48 h post-transfection. Cell lysates of COS-7 cells transfected with plasmids encoding various forms of HA-UL24 were analyzed via Western blotting with an anti-HA antibody (**top panel**). The blot was subsequently stripped and incubated with an antibody against α Tubulin, which served as a loading control (**bottom panel**). The sizes of molecular weight markers are indicated to the left of the panels. Arrows to the right of the panels indicate the positions of HA-UL24 and α Tubulin.

The analysis of the primary sequence of the UL24 C-terminus between aa 241 and 265 suggested a possible “non-classical” nuclear export signal (NES) between aa 250 and 258 that would contain five hydrophobic residues (Figure 3A,B). This sequence was similar to several “non-classical” NES signals reported for other viral proteins such as EIAV and FIV Rev, HCV core, and the PPRSV N-protein. In addition, we identified a putative protein kinase C phosphorylation site [39] between aa 195 and 197 (Figure 3A) using the MotifScan software, and interestingly, the T195 residue has been reported to be phosphorylated in the context of infection [40]. Phosphorylation has been shown to affect the nuclear export of some proteins, for example, Dok-1 [41], a cellular protein that we have previously shown is important for the maintenance of HSV-specific CD8^+^ T cells [42]. To determine whether the T195 phosphorylation site, and the residues I250, L253, F254, V256, and V258, corresponding to a putative NES motif, are important for the localization of UL24 in the cytoplasm, they were replaced with alanines via site-directed mutagenesis to create the mutations T195A, I250A, L253A/F254A, V256A/V258A. Western blotting confirmed that all forms were expressed with the expected size (Figure 3C). 

**Figure 4:**
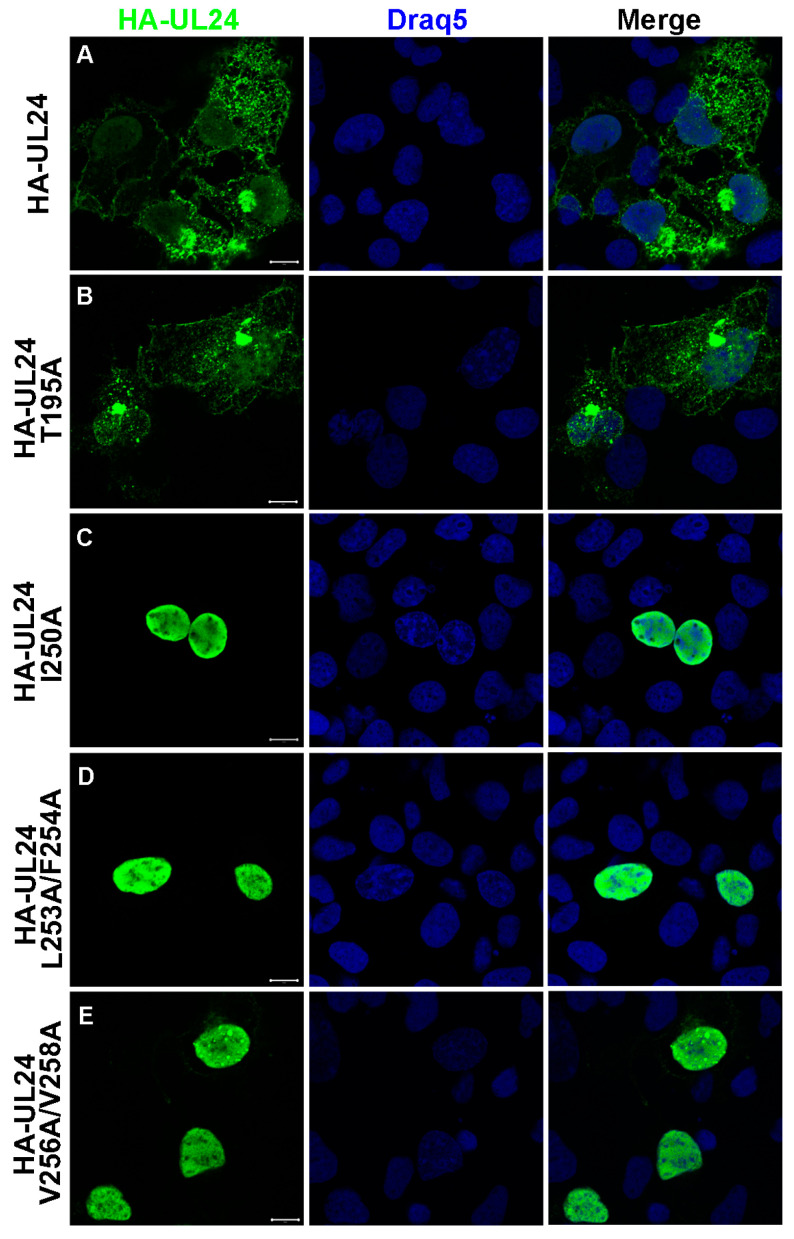
Substitution of hydrophobic residues in the HSV-1 UL24 NES blocks the nuclear export of the protein: (**A**–**E**) Confocal images show the localization of wild-type HA-UL24 and substituted forms with an altered phosphorylation site or NES sequence in transiently transfected COS-7 cells. HA-UL24 was detected via indirect immunofluorescence using a monoclonal antibody directed against HA (green). Nuclei were stained with Draq5 (blue). Merged images are shown in the right-hand panels. Scale bars represent 10 μm.

The localization of the various substituted forms of UL24 was analyzed via indirect immunofluorescence and confocal microscopy (Figure 4). Our results showed that when T195 was replaced with alanine, HA-UL24 was detected in both the nucleus and the cytoplasm, in a manner similar to that seen for the wild-type protein (Figure 4A,B), suggesting that the phosphorylation site T195 does not play a role in the cytoplasmic localization of UL24. In contrast, staining for HA-UL24 variants with the substitutions I250A or L253A/V254A was detected exclusively in the nucleus (Figure 4C,D). Similarly, the form V256A/V258A led to the accumulation of HA-UL24 predominantly in the nucleus with only weak detection in the cytoplasm (Figure 4E). These results demonstrate that these hydrophobic residues are part of a functional NES capable of directing the export of UL24 from the nucleus to the cytoplasm.

### 3.3. Nuclear Export Dependent on the UL24 NES Is Mediated by CRM-1 in Transiently Transfected Cells

**Figure 5:**
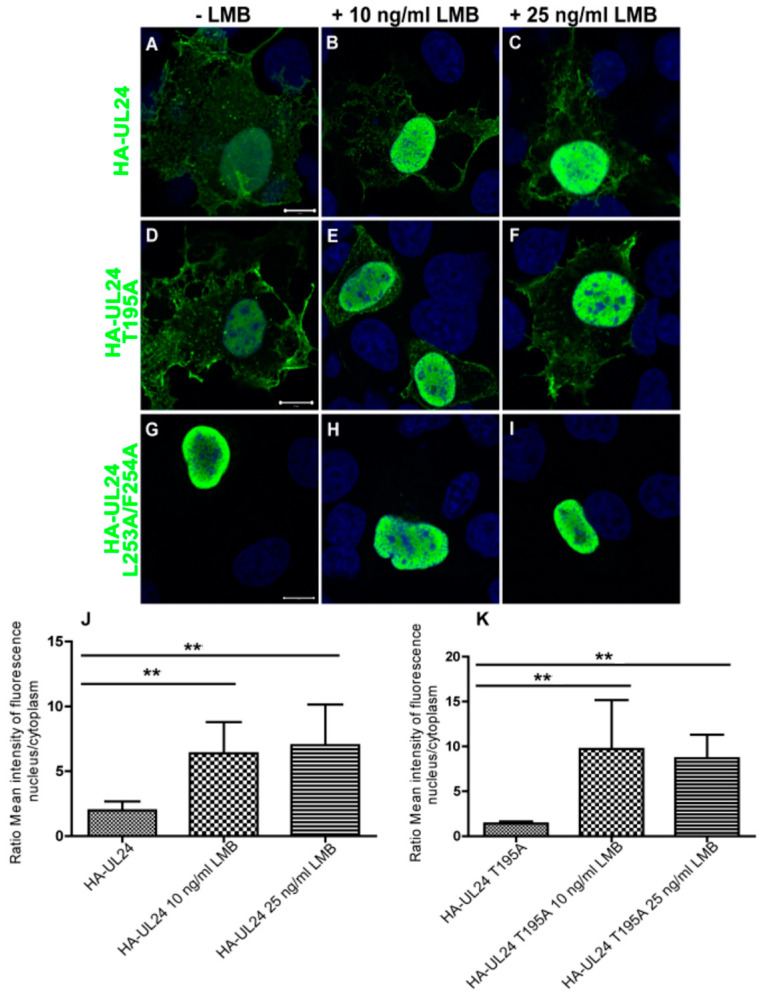
The UL24 NES is sensitive to leptomycin B. COS-7 cells were transiently transfected with a plasmid encoding wild-type HA-UL24 (**A**–**C**) or with plasmids encoding HA-UL24 with an altered phosphorylation site (**D**–**F**) or substituted NES sequence (**G**–**I**). Briefly, 22 h post-transfection, cells were treated for 5 h with 10 ng/mL LMB (**B**,**E**,**H**) or with 25 ng/mL LMB (**C**,**F**,**I**). Representative images acquired using confocal microscopy are shown. The subcellular distribution of HA-UL24 was examined using a monoclonal antibody directed against HA and a secondary antibody conjugated with Alexa-488 (green). Nuclei were stained with Draq 5 (blue). Scale bars represent 10 μm. (**J**,**K**) The quantification of the nuclear–cytoplasmic (N/C) ratio of the mean intensity of fluorescence for HA-UL24 (**J**) and HA-UL24 T195A (**K**) in COS-7 cells in the presence of 10 ng/mL or 25 ng/mL LMB compared with the vehicle alone. The mean N/C ratios were determined from 30 cells over 3 independent experiments. Error bars represent the standard deviation of the mean. **, *p* < 0.0003 compared with control using one-way ANOVA following a Bonferroni correction of the alpha value for multiple comparisons.

To determine whether the nuclear export dependent on the UL24 NES is mediated by CRM-1, we used leptomycin B (LMB), a specific inhibitor of this nuclear export pathway. COS-7 cells were transfected with a plasmid encoding wild-type HA-UL24 or with plasmids encoding the altered forms of HA-UL24. Twenty-two hours later, cells were treated with 10 ng/mL or 25 ng/mL LMB for five hours. We found that the lower concentration of LMB was sufficient to cause the nuclear accumulation of both wild-type HA-UL24 and HA-UL24 T195A (Figure 5A–F). As expected, the treatment of the cells with LMB had no effect on the localization of HA-UL24 with the non-functional L253A/F254A NES, which remained localized in the nucleus under both conditions (Figure 5G–I). The results were quantified by calculating the nuclear–cytoplasmic (N/C) ratio of the mean intensity of fluorescence of HA-UL24 in the presence and absence of LMB. Following the treatment of cells expressing HA-UL24 and HA-UL24 T195A with LMB, the N/C ratio of the mean intensity of fluorescence was significantly higher than that for cells treated with the vehicle alone (Figure 5J–K). Therefore, these results indicate that ectopically expressed UL24 can shuttle between the nucleus and the cytoplasm in a CRM-1-dependent manner.

### 3.4. Mutations Inactivating the UL24 NES Lead to a Syncytial Plaque Phenotype

**Figure 6:**
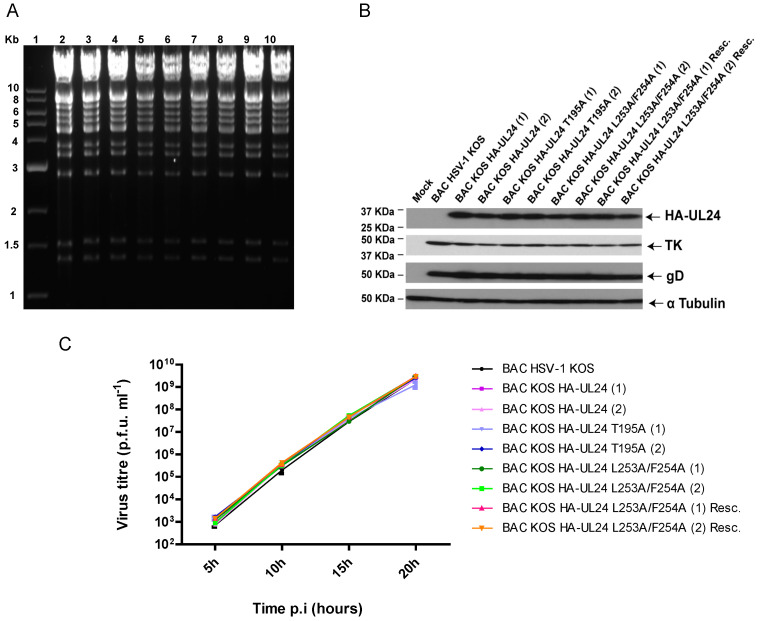
Characterization of recombinant HSV-1 BACs: (**A**) The EcoRV restriction pattern of genomes of the recombinant BAC HSV-1 strains carrying mutations in *UL24*. DNA was resolved on a 0.7% agarose gel and stained with ethidium bromide. The numbers (1) and (2) in the names of the viruses represent the different isolates. Lane 1, 1kb DNA ladder; lane 2, BAC HSV-1 KOS; lane 3, BAC_KOS HA-UL24 (1); lane 4, BAC_KOS HA-UL24 (2); lane 5, BAC_KOS HA-UL24 T195A (1); lane 6, BAC_KOS HA-UL24 T195A (2); lane 7, BAC_KOS HA-UL24 L253A/F254A (1); lane 8, BAC_KOS HA-UL24 L253A/F254A (2); lane 9, BAC_KOS HA-UL24 L253A/F254A (1) Resc.; lane 10, BAC_KOS HA-UL24 L253A/F254A (2) Resc. The sizes of the molecular markers are shown on the left. (**B**) Western blot analysis showing the expression of HA-UL24, HSV-1 TK, and HSV-1 gD by the different recombinant viruses. Lysates of Vero cells mock-infected or infected at an MOI of 10 with the indicated recombinant virus were analyzed via Western blotting with specific antibodies against HA, TK, and gD. The blot was also probed with an antibody against α Tubulin, which served as a loading control. The sizes of molecular weight markers are indicated to the left of the panels. Arrows to the right of the panels indicate the positions of the different proteins. (**C**) Replication curves for the recombinant viruses. Each virus indicated was used to infect Vero cells in duplicate at an MOI of 5. The supernatant and the cell-associated virus were collected at the indicated time points, and the total infectious virus produced was titrated. Error bars represent the standard error of the mean of two independent experiments.

To study the impact of mutations in the C-terminal domain of UL24 on the biology of the virus, we generated various mutants of HSV-1 harboring several of the substitutions analyzed in our transfection experiments. For this purpose, we used the two-step Red recombination method to mutate the KOS genome cloned as a bacterial artificial chromosome as described previously [36,37,38]. The recombinant BACs generated expressed UL24 with an N-terminal HA tag, which allowed for its easy detection via indirect immunofluorescence and confocal microscopy. The N-terminal insertion of the HA tag in the UL24 ORF has been shown previously not to alter viral replication [14]. The mutation L253A/F254A targeting the NES, and the mutation T195A targeting the phosphorylation site of UL24 were inserted into BAC-HSV-1 KOS; two independent isolates (i.e., from two independent transfections) were produced for each recombinant virus. A rescue virus for L253A/F254A was also constructed. To confirm that the recombinant BACs had not undergone any major genomic rearrangements, BAC DNA with the *UL24* mutations was investigated by restriction enzyme analysis. All mutants generated showed EcoRV digestion patterns similar to that of wild-type BAC HSV-1 KOS (Figure 6A). These BAC DNAs were transfected into Vero cells for the production of recombinant viruses. The expression of HA-UL24 was verified using Western blot analysis of Vero cell lysates harvested 18 hpi (Figure 6B). HA-UL24 levels were similar for all of the viruses. The expression of TK was also confirmed to be similar for all the strains. The membrane was stripped and reprobed with an antibody against α Tubulin, which served as a loading control (Figure 6B).

Virus yield was tested in a one-step replication assay (Figure 6C). Vero cells were infected at an MOI of 5, and at the indicated time points, the total virus (cell-free and -associated) was harvested and titrated. We observed that all of the viruses tested replicated similarly to BAC HSV-1 KOS. Thus, we concluded that the residues substituted in the C-terminal domain of UL24 did not affect HSV-1 yield in cell culture.

**Figure 7:**
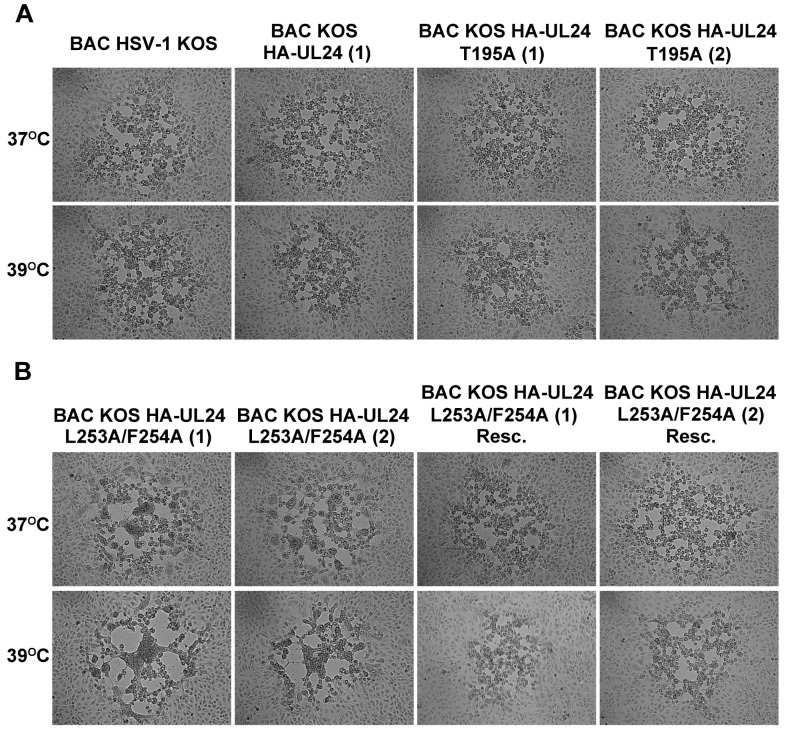
Mutations targeting the UL24 NES result in syncytial plaques. Representative plaques observed at 37 °C and 39 °C on Vero cells: (**A**) Plaques produced by the viruses BAC HSV-1 KOS, BAC_KOS HA-UL24 (1), and BAC_KOS HA-UL24 T195A (1) and (2). (**B**) Plaques produced by the viruses BAC_KOS HA-UL24 L253A/F254A (1) and (2) and BAC_KOS HA-UL24 L253A/F254A (1) Resc and (2) Resc.

Many UL24 mutant viruses form syncytial plaques, a phenotype that is more penetrant at 39 °C as described previously [4]. Therefore, we studied the plaque phenotypes produced by the different recombinant viruses. Vero cells were infected at low MOI and incubated at 37 °C or 39 °C for two days. Plaque morphology was visualized using an inverted phase contrast microscope. At 37 °C, the plaques formed by BAC KOS HA-UL24 (1) and (2), BAC KOS HA-UL24 T195A (1) and (2), and BAC KOS HA-UL24 L253A/F254ARescue were similar to those formed by the BAC HSV-1 KOS; however, we noted small syncytia for BAC KOS HA-UL24 L253A/F254A (Figure 7A,B, top panels). At 39 °C, we observed that both isolates of BAC_KOS HA-UL24 L253A/F254A formed large syncytia. In contrast, BAC_KOS HA-UL24, BAC_KOS HA-UL24 T195A, and the rescue viruses formed non-syncytial plaques at 39 °C (Figure 7A,B, bottom panels). The plaques formed by BAC_KOS HA-UL24 L253A/F254A were similar to those formed by the UL24-null virus, UL24X [4]. These results demonstrate that a virus encoding a UL24 protein with a non-functional NES expresses a syncytial plaque phenotype similar to that observed for a virus that does not express UL24.

### 3.5. The HSV-1 UL24 Protein Shuttles between the Nucleus and the Cytoplasm during Infection

**Figure 8:**
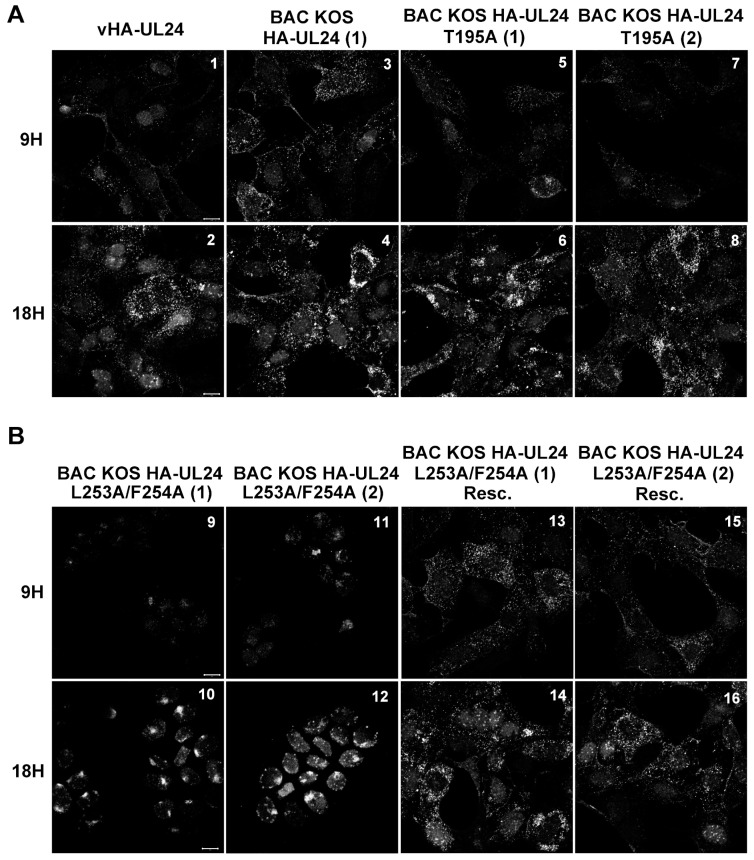
Mutations targeting the UL24 NES block nuclear export of UL24 during infection. Vero cells grown on coverslips were infected with the indicated virus at an MOI of 10. At 9 and 18 hpi; the cells were washed, fixed, and immunostained; and representative images were acquired using confocal microscopy. The intracellular distribution of HA-UL24 was examined using a monoclonal antibody directed against HA and a secondary antibody conjugated with Alexa-488. (**A**) vHA-UL24, BAC_KOS HA-UL24 (1), BAC_KOS HA-UL24 T195A (1), BAC_KOS HA-UL24 T195A (2); (**B**) BAC_KOS HA-UL24 L253A/F254A (1), BAC_KOS HA-UL24 L253A/F254A (2); BAC_KOS HA-UL24 L253A/F254A (1) Rescue; BAC_KOS HA-UL24 L253A/F254A (2) Rescue. Scale bars represent 10 μm.

We investigated the subcellular distribution of HA-UL24 protein following infection by the different recombinant viruses (Figure 8). Vero cells were infected with the indicated virus at an MOI of 10. At 9 and 18 hpi, cells were fixed, and the subcellular localization of HA-UL24 was analyzed via indirect immunofluorescence and confocal microscopy. At both 9 and 18 hpi, we observed that UL24 localized in the nucleus and the cytoplasm with some nucleolar staining in BAC_KOS HA-UL24 (1)-infected cells in a manner similar to that observed previously for vHA-UL24 [14] (Figure 8A, panels 1,3). At 18 hpi, HA-UL24 was distributed in the nucleus and the cytoplasm with prominent perinuclear staining (Figure 8A, panels 1–4). Similar patterns were observed at 9 and 18 hpi in cells infected with either isolate of BAC_KOS HA-UL24 T195A (Figure 8A, panels 5–8). In contrast, HA-UL24 remained entirely nuclear in cells infected with either isolate of BAC_KOS HA-UL24 L253A/F254A, which encodes a UL24 protein with an altered NES sequence (Figure 8B, panels 9–12). In cells infected with the rescue viruses, HA-UL24 was distributed in the nucleus and the cytoplasm (Figure 8B, panels 13–16) in a manner similar to that observed for the BAC_KOS HA-UL24 (Figure 8A, panels 3–4). These results indicate that, during infection, HA-UL24 shuttles between the nucleus and the cytoplasm due to the presence of the NES we identified in the C-terminal domain of the protein.

**Figure 9:**
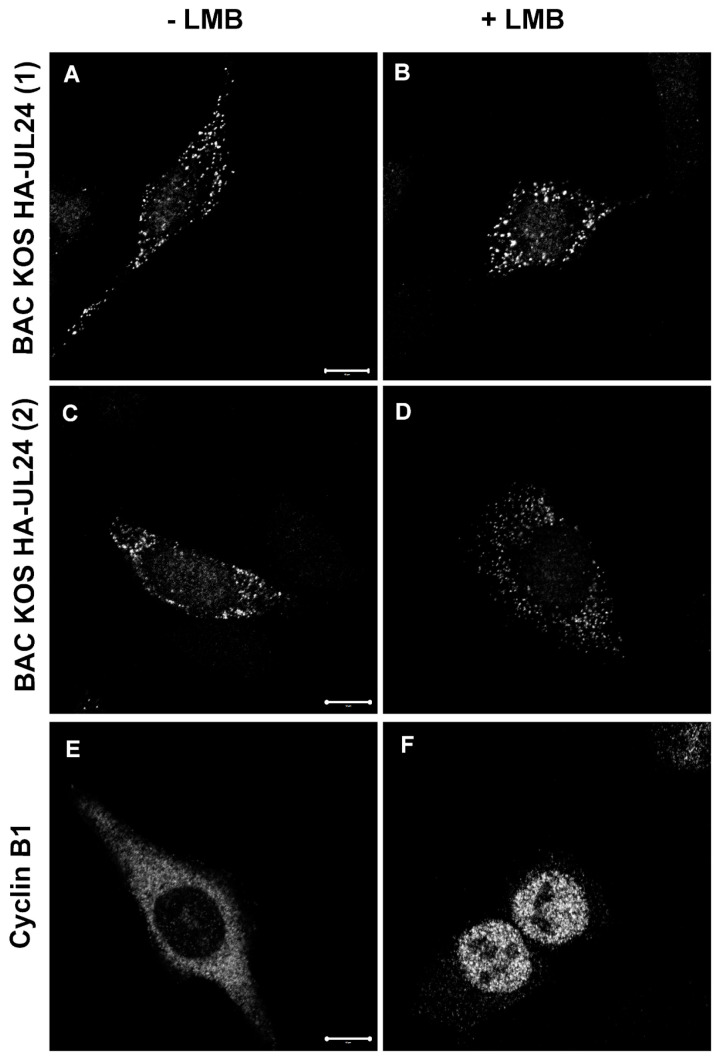
CRM-1 treatment does not block the nuclear export of UL24 in infected cells. HeLa cells were mock-infected (**E**,**F**) or infected with either BAC_KOS HA-UL24 (1) (**A**,**B**) or BAC_KOS HA-UL24 (2) (**C**,**D**) at an MOI of 10. At 8 hpi, the cells were treated for 5 h with 20 ng/mL LMB (**B**,**D**,**F**). Cells were washed, fixed, and immunostained, and representative images were acquired using confocal microscopy. The intracellular distribution of HA-UL24 was examined using a monoclonal antibody directed against HA and a secondary antibody conjugated to Alexa-488 (**A**–**D**). The detection of the cyclin β1 protein was considered a positive control for NES sensitivity to LMB (**E**,**F**). Scale bars represent 10 μm.

To determine whether the nuclear export of HSV-1 UL24 is mediated by CRM-1 during infection, HeLa cells were infected with either isolate of BAC_KOS HA-UL24. At 8 hpi, cells were either treated with vehicle alone or treated with 20 ng/mL LMB for 5 h (Figure 9A–D). To validate our experiment, mock-infected HeLa cells were similarly treated with LMB and processed for indirect immunofluorescence with a monoclonal antibody directed against Cyclin β1, which contains a CRM-1-dependent NES [43] (Figure 9E,F). Although LMB blocked the nuclear export of HA-UL24 in transient transfection assays (Figure 5), the treatment of infected cells with LMB had no effect on the localization of HA-UL24 protein (Figure 9A–D). In contrast, LMB inhibited the nuclear export of Cyclin β1, which was used as a positive control (Figure 9E,F). These results suggest that the nuclear export of HA-UL24 is either mediated by an export pathway other than CRM-1 during infection or that there is redundancy with other export pathways in the infected cells.

### 3.6. The UL24-Mediated Redistribution of Nucleolin Does Not Depend on the Ability of UL24 to Shuttle between the Nucleus and the Cytoplasm

**Figure 10:**
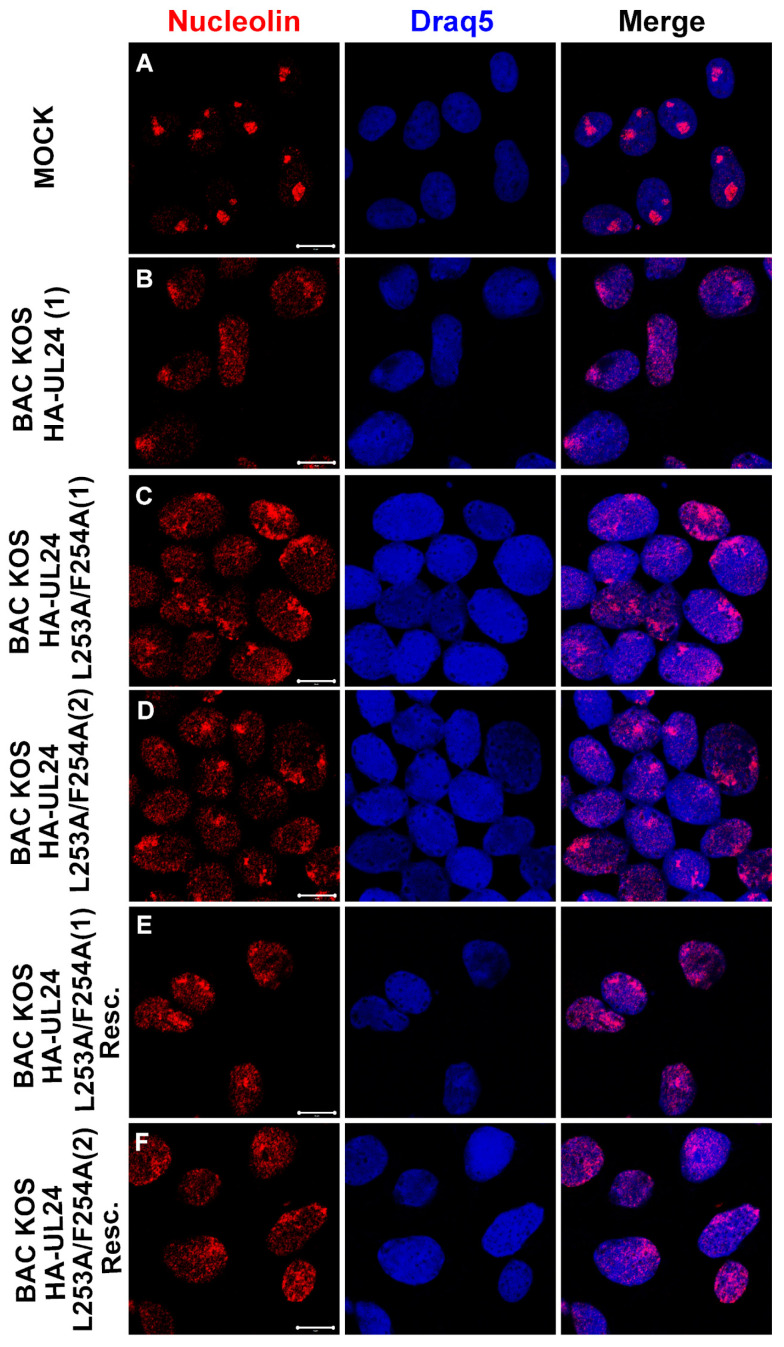
Mutations inactivating the NES of UL24 do not inhibit the redistribution of nucleolin in infected cells. Confocal images of mock-infected Vero cells (**A**), or cells infected with the indicated recombinant viruses (**B**–**F**) at an MOI of 10. At 18 hpi, cells were washed, fixed, and immunostained for nucleolin (red). Nuclei were stained with Draq5 (blue). Merged images are shown in the right-hand panels. Scale bars represent 10 μm.

One of the known activities of UL24 is to induce the dispersal of nucleolin from nucleoli throughout the nucleoplasm [8,14]. It is also known that nucleolin can shuttle between the nucleus and the cytoplasm and that it can associate with the plasma membrane via its glycine–arginine-rich (GAR) domain [44,45,46]. Therefore, we investigated whether the nuclear export of UL24 plays a role in the dispersal of nucleolin. Vero cells were grown on coverslips and either mock-infected or infected with BAC_KOS HA-UL24 (1), BAC_KOS HA-UL24 L253A/F254A (1) and (2), or the rescue viruses. At 18 hpi, cells were fixed, and the distribution of nucleolin was analyzed via indirect immunofluorescence and confocal microscopy (Figure 10A–F). As expected, nucleolin staining was almost exclusively nucleolar in mock-infected cells. In contrast, both isolates of BAC_KOS HA-UL24 L253A/F254A, which encode an altered NES sequence, retained the ability to disperse nucleolin throughout the nucleoplasm similar to that observed for BAC_KOS HA-UL24 and the rescue viruses (Figure 10B–F). These results indicate that the redistribution of nucleolin does not depend on the ability of UL24 to shuttle between the nucleus and the cytoplasm.

## 4. Discussion

Based on the previously observed differential localization of the N- and C-terminal domains of HSV-1 UL24, we carried out a structure–function analysis of the C-terminus of the protein, with the goal of elucidating its role in infection. We generated a series of mammalian expression vectors encoding UL24 of HSV-1 with deletions in the C-terminal domain. In the study of the subcellular localization of these truncated versions of UL24, the forms HA-UL24Stop197, HA-UL24Stop219, and HA-UL24stop265 were found both in the nucleus and in the cytoplasm. In contrast, for HA-UL24Stop252 and HA-UL24Stop240, pronounced staining was only detected in the nucleus. This study revealed that the region between amino acids 241 and 265 was critical for the cytoplasmic localization of the HSV-1 UL24 protein. According to the analysis results of the primary sequence of UL24, the region between the amino acids 250 and 258 (IAALFCVPV) is consistent with an NES sequence. It is possible that elements within the C-terminal domain affect the stability of the protein because we observed increased steady-state levels of expression for the forms HA-UL24Stop265, HA-UL24Stop252, HA-UL24Stop240, and HA-UL24Stop219 in comparison to HA-UL24 stop197 and wild-type HA-UL24; however, expression levels did not correlate with a specific localization of the protein. The reduction in the cytoplasmic accumulation of the protein for HA-UL24Stop252 and HA-UL24Stop240 can be explained by the loss of the NES that we identified (AA 250-258); however, the restoration of cytoplasmic localization for the Stop219 and Stop197 forms of UL24 was unexpected. When expressed alone, the C-terminal domain is cytoplasmic even though a bioinformatic study has identified a bipartite NLS extending from aa199 to aa 229 [8]. This NLS is partially and fully lost in Stop219 and Stop197, respectively. Thus, one explanation for the restoration of cytoplasmic accumulation for Stop 219 and 197 is that, in the absence of both the NES and NLS, the small polypeptides expressed (24 kDa and 22 kDa, respectively) can move across the nuclear membrane through passive diffusion because they are below the molecular weight cutoff for active transport through the nuclear pores. But because the N-terminal region of UL24 localizes to the nucleus, some form of a cytoplasmic retention domain is likely present in the Stop197 and Stop219 forms of UL24 but is absent in the N-terminal UL24 construct we have used previously, which ends at residue 192.

The directed mutagenesis of the residue T195 (targeting the phosphorylation site) had no effect on the cytoplasmic localization of the protein. In contrast, the targeted mutagenesis of the residues predicted to form an NES (I250, L253, F254, V256, and V258) significantly increased the nuclear localization of UL24, confirming the functionality of the NES identified in the C-terminus of UL24. Moreover, residues within the NES are conserved among orthologs of UL24 such as UL24 of HSV-2 and UL24 of BoHV-1 and 2, and they likely play a role in the biology of these herpesviruses as well. These same residues were important for the localization of the UL24 protein in the cytoplasm during infection. The UL24.5 protein that is expressed from one of the shorter *UL24* transcripts and has as a start codon the equivalent of residue M122 in the full-length UL24 ORF [47], would retain the NES. We found that in cells infected with NES mutants (BAC HSV-1 HA-UL24 L253A/F254A (1) and (2)), HA-UL24 was only detected in the nucleus. Our observations suggest that the UL24 protein is able to shuttle between the nucleus and the cytoplasm during infection. We sometimes noticed that the distribution of UL24 appeared as multiple spots, particularly in the context of a block in nuclear export. This may be a result of the overexpression of the protein in the nucleus, leading to the formation of nuclear aggresomes as observed for the human cytomegalovirus ortholog UL76 [48].

Although in transient transfection experiments, we found that the nuclear export of UL24 was dependent on CRM-1, the nuclear export of UL24 was CRM-1-independent in the context of infection. A similar dichotomy has also been observed for other HSV proteins such as ICP27 and Us3 [49,50,51]. Previous studies have demonstrated interactions of proteins containing an NES with export receptors other than CRM-1, such as exportin 2 [52], exportin 4 [53], exportin 5 [54], and the mRNA export factor TAP [55,56,57]. Thus, it is possible that one of these alternative receptors allows for the nuclear export of HSV-1 UL24 during infection, or there may be redundancy between CRM-1 and another export pathway. Also, we cannot rule out that, during infection, UL24 interacts with other viral and/or cellular proteins that may mediate the nuclear export of UL24 indirectly. 

The NES-dependent nuclear export of certain viral proteins is important for viral replication efficiency, as has been shown for example for HIV REV [58,59] and HCV core [33] proteins. Previous studies have shown that a UL24X virus has a replication defect in cell culture and in vivo [12]. While we found that a mutation targeting the NES of UL24 (i.e., L253A/F254A) did not cause a decrease in viral yield, it led to an altered plaque phenotype with the formation of syncytial plaques similar to those observed for the UL24X virus. This is a similar phenotype to the phenotype we have observed for one other *UL24* mutation, G121A [6]. Since the C-terminal domain of UL24 localizes to the cytoplasm, and UL24 is a leaky-late protein, one could hypothesize that it is involved in viral envelopment and release, which require membrane fusion. Our previous report showing the impact of a UL24-null mutation on the cytoplasmic distribution of HSV-1 glycoproteins involved in fusion [17] is consistent with a block in the nuclear export of UL24 leading to a syncytial phenotype, and may also be related to our previous observation that, when expressed alone, the C-terminal domain of UL24 localizes to the *trans*-Golgi [8]. The viruses BAC_KOS HA-UL24 L253A/F254A (1) and (2), with mutations that destroy the NES such that the UL24 protein is found exclusively in the nucleus, will be useful tools for elucidating the molecular mechanisms of the formation of syncytial plaques without the confounding effects of the reduced viral replication observed with UL24-null viruses. 

UL24 is necessary and sufficient to drive the redistribution of nucleolin from the nucleolus into the nucleoplasm [8,14]. Nucleolin shuttles between the nuclear and cytoplasmic compartments and is also found at the plasma membrane [60]. Cell surface nucleolin has been identified as a receptor for several viruses [61,62]. These characteristics of nucleolin have prompted the hypothesis that the syncytial plaque phenotype seen with UL24-deficient viruses is due to a block in the redistribution of nucleolin. However, we found that blocking the shuttling ability of UL24 did not hinder the ability of UL24 to induce the redistribution of nucleolin but did cause a syncytial plaque phenotype. Thus, this result does not support a model in which the loss of the nucleolar dispersal of nucleolin seen for UL24-null viruses is the cause of syncytia formation. 

In conclusion, we identified a functional NES in the HSV-1 UL24 protein. A mutation inactivating this NES revealed that the nuclear export of UL24 is necessary for a wild-type plaque phenotype. Future studies will be aimed at further probing the impact of the NES mutation in the context of viral infection, particularly in the latter stages of viral morphogenesis in the cytoplasm.

## Figures and Tables

**Table 1:** Primers used for cloning of mutated forms of HSV-1 UL24 with C-terminal deletions.

Name	Sequence 5′ → 3′ *^a^*
UL24 5′BglIIF	CCC**AGATCT**GCGGCACGCTGTTG
UL24CtermStop795R	CACA**GTCGAC**TCAGTCAGTCATCGGGGTTTGGTCTTGGTGG
UL24CtermStop756R	CACA**GTCGAC**TCAGTCAGTCACGCCGCGATCCTCTTAAG
UL24CtermStop720R	CACA**GTCGAC**TCAGTCAGTCAGGAGGCGGGGGTTTGGTC
UL24CtermStop657R	CACA**GTCGAC**TCAGTCAGTCACCCAGAGTGTGACCTTTTCGG
UL24CtermStop591R	CACA**GTCGAC**TCAGTCAGTCATCGCTGGGTCCTAGGCTCC

***^a^*** Restriction sites for cloning are shown in bold, and stop codons are underlined.

**Table 2:** Primers used for site-directed mutagenesis of HSV-1 UL24.

Name	Sequence 5′ → 3′ *^a^*	Mutation *^b^*
UL24_T195Atop	CCCATGGAGCCTAGG**G**C**A**CAGCGAGCCCGTCGC	
botUL24_T195A	GCGACGGGCTCGCTG**T**G**C**CCTAGGCTCCATGGG	T195A
UL24_L253A-F254Atop	AAGAGGATCGCGGCG**GC**C**GC**CTGCGTGCCCGTGGC	
botUL24_L253A-F254A	GCCACGGGCACGCAG**GC**G**GC**CGCCGCGATCCTCTT	L253A–F254A
UL24_V256A-V258Atop	GCTCTTCTGCG**C**GCCCG**C**GCGCACCAAGA	
botUL24_V256A-V258A	TCTTGGTGGCC**G**CGGGC**G**CGCAGAAGAGC	V256A–V258A

***^a^*** The mutated nucleotides are shown in bold. ***^b^*** Each mutation is named by the amino acid of the wild-type sequence, followed by the position of the mutation and the final residue after mutagenesis.

**Table 3:** Primers used for the generation of recombinant viruses by the BAC system.

Name	Sequence 5′→ 3′ *^a^*	Mutation
BAC_HA_UL24_F BAC_HA_UL24_R	TCCGTGGCTTCTTGCTGCCGGCGAGGGCGCAACGCCGTA CGTCGGTTGCT**ATGTACCCATACGATGTTCCAGATTACG** **CT**GCCGCGAGAACGCGCAGCCTGGTCGAGGATGACGAC GATAAGTA CGTACCCCTGCCATCAACACGCGTCTGCGTTCGACCAGG CTGCGCGTTCTCGCGGC**AGCGTAATCTGGAACATCGTA** **TGGGTACAT**AGCAACCGACGTACGGCGTTCAACCAATT AACCAATTCTGA	Insertion of N-terminal HA tag in UL24
BAC_HSV1T195A_F BAC_HSV1T195A_R	CGTGCGGATGCTCCAGAGCCTGTCCACGTATACGGTCCC CATGGAGCCTAGG**G**C**A**CAGCGAGCCCGTCGCCGCCGAG GATGACGACGATAAGTA CTGCTCGCAGACCCCCGGGCAGCGCCGCCGCGGCGGCG ACGGGCTCGCTG**T**G**C**CCTAGGCTCCATGGGGACCGTA**C**A ACCAATTAACCAATTCTGA	UL24 mutation T195AA
BAC_HSV1L253A-F254A_F BAC_HSV1L253A-F254A_R RescueBAC_HSV1L253A-F254A_F RescueBAC_HSV1L253A-F254A_R	CAAACCCCCGCCTCCACGGAGGGCGGGGGGGTGCTTAA GAGGATCGCGGCG**GC**C**GC**CTGCGTGCCCGTGGCCACCA AAGGATGACGACGATAAGTA CTCTCATTCGGAGGCAGCTCGGGGTTTGGTCTTGGTGGCC ACGGGCACGCAG**GC**G**GC**CGCCGCGATCCTCTTAAGCAC AACCAATTAACCAATTCTGA CAAACCCCCGCCTCCACGGAGGGCGGGGGGGTGCTTAA GAGGATCGCGGCG**CT**C**TT**CTGCGTGCCCGTGGCCACCAA AGGATGACGACGATAAGTA CTCTCATTCGGAGGCAGCTCGGGGTTTGGTCTTGGTGGCC ACGGGCACGCAG**AA**G**AG**CGCCGCGATCCTCTTAAGCAC AACCAATTAACCAATTCTGA	UL24 mutation L253AF254A Reinsertion of wild type UL24 sequence

***^a^*** Nucleotides in bold represent point mutations, and underlined nucleotides are those designed to anneal to the kanamycin resistance gene.

## Data Availability

Data are contained within the article.
